# Identification and Characterization of *Troponin T* Associated with Development, Metabolism and Reproduction in *Tribolium castaneum*

**DOI:** 10.3390/ijms26062786

**Published:** 2025-03-19

**Authors:** Wenzhuo Li, Yaning Sun, Yuanye Liang, Yifan Wang, Yongmei Fan, Mengmeng Li, Ranfeng Sun, Jia Xie

**Affiliations:** Key Laboratory of Green Prevention and Control of Tropical Plant Diseases and Pests, Ministry of Education, School of Tropical Agriculture and Forestry, Hainan University, Danzhou 570228, China; 22220951320026@hainanu.edu.cn (W.L.); 23220951320050@hainanu.edu.cn (Y.S.); 24220951320142@hainanu.edu.cn (Y.L.); 22220951320038@hainanu.edu.cn (Y.W.); yongmeifan@126.com (Y.F.); 222209513200246@hainanu.edu.cn (M.L.)

**Keywords:** *troponin T*, RNA interference, metamorphosis, physiological disorder, insect hormones

## Abstract

As a tropomyosin-binding component, troponin T (TnT) is essential for the Ca^2+^ regulation of striated muscles’ contraction and locomotion activity, but its impacts on the growth and development of insects have rarely been reported. In this study, *TnT* was identified and functionally characterized in *Tribolium castaneum* by RNA interference (RNAi) and transcriptome analysis. The *TnT* of *T. castaneum* contained a 1152 bp open reading frame encoding 383 amino acids. It displayed the highest expression in late pupae and was highly expressed in the integument and CNS. Both the larval and early pupal injection of ds*TnT* led to 100% cumulative mortality before the pupal–adult transition. Late pupal RNAi caused 26.01 ± 4.29% pupal mortality; the survivors successfully became adults, but 49.71 ± 6.51% died in 10 days with a dried and shriveled abdomen, poorly developed reproductive system and no offspring. Additionally, RNA sequencing results indicated that key ecdysteroid and juvenile hormone biosynthesis genes (*CYP314A1*, *aldehyde dehydrogenase family 3 member B1* and *farnesol dehydrogenase*) were affected, as well as several cuticle protein, nutrition metabolism and immune-related genes, suggesting that *TnT* may play prominent roles in development, metabolism and reproduction by affecting these pathways. This study could provide a brand-new target gene in the RNAi strategy for pest control.

## 1. Introduction

Troponin, located in the thin filaments, plays an essential regulatory role in muscle contraction and relaxation [1]. It contains three subunits: the Ca^2+^-binding subunit troponin C (TnC), the inhibitory subunit troponin I (TnI), and the tropomyosin-binding subunit troponin T (TnT) [2]. Among them, TnT serves as the sarcomeric thin filament anchoring subunit of the troponin complex, which anchors TnI and TnC to tropomyosin, thereby playing an organizer role in transducing Ca^2+^ signals in the regulation of muscle contraction [3]. The emergence of TnT can be traced to approximately 700 million years ago in bilateral symmetric invertebrate animals [4]. Vertebrate and invertebrate TnTs have both conserved core structures and significantly diverged structures, reflecting that they may have conserved functions in regulating muscle contraction and differentiated muscle type and species-specific adaptations [5].

Vertebrates have evolved with three homologous genes encoding the slow skeletal muscle, cardiac and fast skeletal muscle type isoforms of *TnT*, named *TnnT1*, *TnnT2* and *TnnT3*, respectively [6]. These genes have been intensively investigated in the human medical field, since some serious diseases are typically caused by their mutations. As a predictor of mortality in patients attending the emergency department with atrial fibrillation, *TnnT2* (cardiac *TnT*) plays a major role in the evaluation of myocardial injury and the prediction of cardiovascular outcomes for cardiac and non-cardiac causes [7]. A mutation in *TnnT1* could cause nemaline body myopathy, while distal arthrogryposis has been identified as potentially being caused by a mutation in *TnnT3* [8,9]. The structure and function of the vertebrate *TnT* also exhibit certain differentiations due to species-specific adaptation. For instance, multiple avian-specific isoforms caused by alternative splicing may have contributed to them adapting to activities such as flying and hunting [10], while the toad heart has exclusively utilized the slow skeletal muscle *TnnT1* for higher contractile and relaxation velocities to adapt to living on land [11].

Most invertebrates, such as insects, have only one *TnT* gene [12]. The research on *TnT* in insects lags far behind that in vertebrates, mainly focusing on its role in muscle function, locomotor mechanics and other relevant activities. A *Drosophila melanogaster TnT* mutant has been found that specifically affects indirect flight muscles [13]. Similarly, the alternative splicing of *TnT* was utilized to modulate the calcium sensitivity of the flight muscles in *Libellula pulchella*, thereby achieving ecologically relevant variations in flight performance and energy consumption [14]. In addition, *TnT* has also been found to be related to performing the normal physiological functions of mandibular muscles in ants, maintaining weight and nutrition in moths, participating in the regulation of metabolic diseases in dragonflies, and promoting wing expansion and maintaining genitalia morphology in cockroaches [15,16,17].

However, most analyses on the function of *TnT* genes have been based on deciphering their alternative splicing patterns. As one of the techniques for exploring gene function, RNA interference (RNAi) has promising application prospects in pest control and insecticide target screening. At present, an RNAi analysis of insect *TnT* has only been performed in *Periplaneta americana*, an incomplete metamorphosis insect, leaving gaps in the knowledge of its role in complete metamorphosis insects, as well as related regulatory mechanisms [15]. In this study, the *TnT* gene in *Tribolium castaneum*, a powerful insect model for RNAi-based genetics research, was identified, and its functions were characterized by RNAi. We clarified its crucial role in larval survival, pupal–adult metamorphosis, tissue morphology and egg production in *T. castaneum*. More importantly, transcriptome sequencing was performed to reveal the possible mechanism of *TnT*. Our work provides a comprehensive molecular characterization of *TnT* that can further yield uniquely instructive insights into pest control.

## 2. Results

### 2.1. Sequence Identification and Phylogenetic Analysis

**Figure 1:**
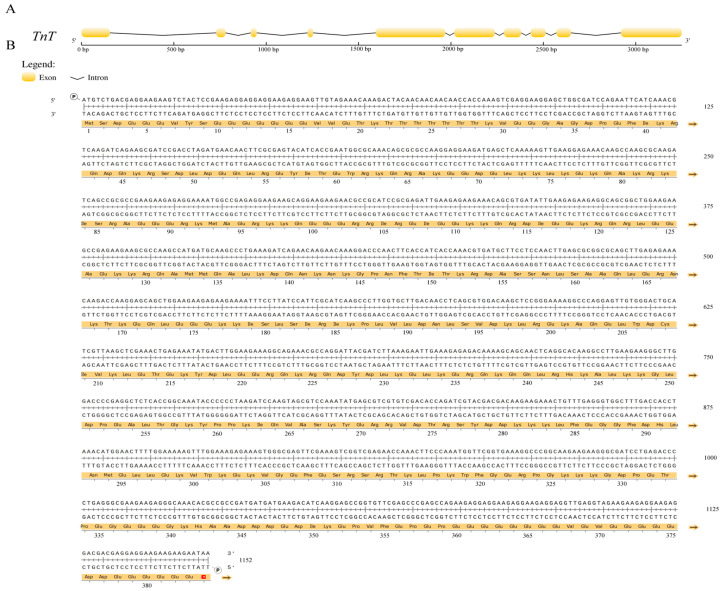
(**A**) The *TnT* gene structure of *T. castaneum*. (**B**) The *TnT* gene sequence of *T. castaneum*. The termination codon TAA was annotated with an asterisk (*).

**Figure 2:**
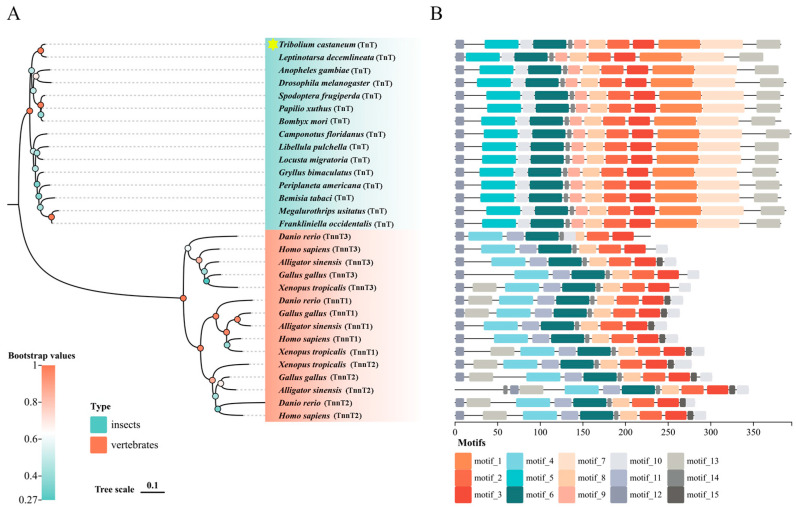
(**A**) Phylogenetic analysis of TnT proteins from different species. The protein sequences were aligned using MEGA11, and the tree was constructed using the neighbor-joining method with 1000 bootstrappings. *T. castaneum* was annotated with a yellow star. (**B**) Motif analysis of TnT proteins from different species.

A total of 30 *TnT* genes in 15 insects and 5 vertebrates were derived from the genome databases in NCBI. The *TnT* gene of *T. castaneum*, which was verified to be correct, contained a 1152 bp open reading frame encoding a protein of 383 amino acids (Figure 1). Only one *TnT* gene existed in the insects, and phylogenetic analysis indicated that the *TnT* genes of insects and vertebrates possessed the same origin and the gene had duplicated to three homologs in the vertebrates (Figure 2A). Motif analysis revealed that TnT genes in vertebrates and insects shared seven identical motifs (motifs 2–4, 6, 8, 10, 12), and the insects had four specific motifs (motifs 1, 5, 7 and 9), which may reflect differential functional adaptations (Figure 2B).

### 2.2. Temporal and Spatial Expression Patterns of TnT

**Figure 3:**
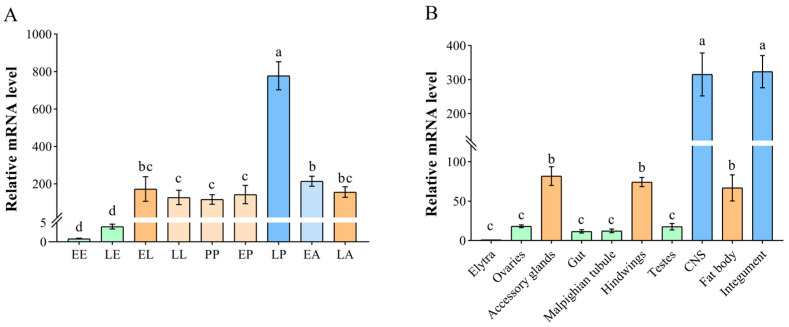
The temporal and spatial expression patterns of *TnT* in *T. castaneum*. (**A**) The different developmental stages are EE: early eggs; LE: late eggs; EL: early larvae; LL: late larvae; PP: pre-pupae; EP: early pupae; LP: late pupae; EA: early adults; and LA: late adults. (**B**) The various tissues of late adults are the elytra, ovaries, accessory glands, gut, Malpighian tubule, hindwings, testes, CNS, fat body, and integument. The cDNA template of *Tribolium ribosomal protein S3* (*rps3*) served as an internal control. The lowercase letters above the columns represent statistically significant differences (*p* < 0.05).

At all developmental stages, *TnT* exhibited the lowest expression level in the embryonic stage and displayed relatively high levels at other stages, especially in the LP stage (Figure 3A). Moreover, it had the highest expression in the CNS and integument, followed by the accessory glands, hindwings and fat body, while it displayed low expression in the elytra, ovaries, gut, Malpighian tubule and testes (Figure 3B).

### 2.3. RNAi Phenotypes of TnT in T. castaneum

**Figure 4:**
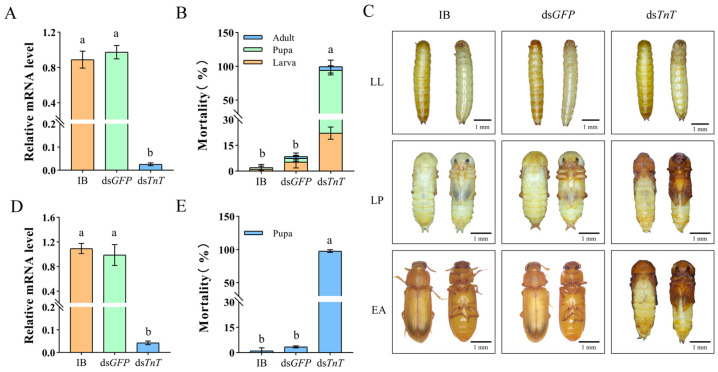
(**A**) Efficiency of larval RNAi. (**B**) Mortality of the beetles after larval RNAi. (**C**) Larval RNAi resulted in lethal phenotypes in late larvae (LL), late pupae (LP) and early adults (EA). (**D**) Efficiency of early pupal RNAi. (**E**) Mortality of the beetles after early pupal RNAi. IB, buffer injection; ds*GFP*, ds*GFP* injection; ds*TnT*, ds*TnT* injection. The lowercase letters above the columns represent statistically significant differences (*p* < 0.05).

**Figure 5:**
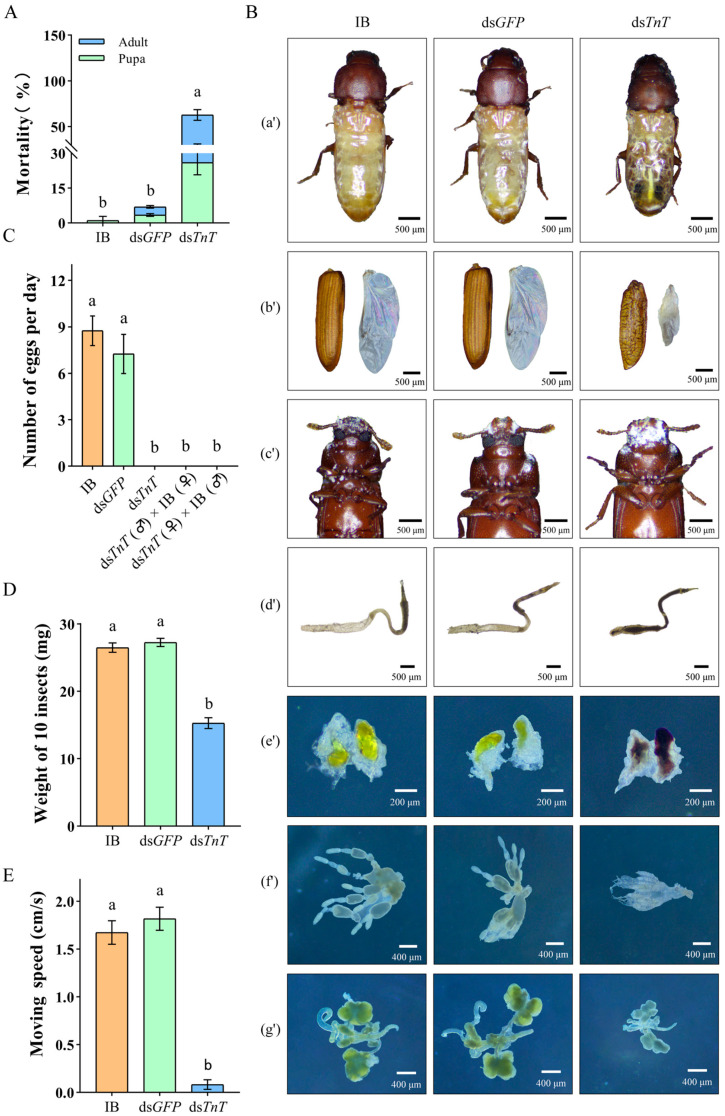
(**A**) Mortality of beetles after late pupal RNAi of *TnT*. (**B**) Late pupal RNAi resulted in abnormalities in various tissues. (a’) Dried and shriveled abdomen; (b’) irregularly wrinkled elytra and hindwings; (c’) mouthparts coated with flour; (d’) gut filled with flour; (e’) black-red, chunky fat body containing abnormal adipocytes, found in abdomen of ds*TnT* adults; (f’) poorly developed ovaries dissected from ds*TnT* female adults; (g’) poorly developed accessory glands and testes dissected from ds*TnT* male adults. (**C**) Average egg numbers of IB, ds*GFP* and ds*TnT* beetles after late pupal RNAi of *TnT*. (**D**) Average weight of 10 beetles in different groups. (**E**) Average moving speed of beetles in different groups. The lowercase letters above the columns represent statistically significant differences (*p* < 0.05).

The late larval RNAi of *TnT* successfully suppressed the target gene (Figure 4A), which caused 100% cumulative mortality during the process of the larval–pupal–adult transition (Figure 4B). The early pupal RNAi of *TnT* also successfully inhibited the target gene’s expression (Figure 4D) and caused 100% defects in pupal–adult metamorphosis (Figure 4E). Additionally, when late pupal RNAi was performed, only 26.01 ± 4.29% of the pupae were arrested at the time of eclosion. The remain individuals could successfully enter the adult stage, but had a mortality of 49.71 ± 6.51% in 10 days (Figure 5A). The survivors had dried and shriveled abdomens containing a black-red, lumpy fat body with abnormal adipocytes and irregularly wrinkled wings, and their mouthparts were coated with flour, and their guts were filled with flour (Figure 5B). Moreover, the reproductive system of the ds*TnT* adults was poorly developed (Figure 5B), and they were completely unable to lay eggs (Figure 5C). They also had a significantly decreased body weight (Figure 5D) and exhibited a lower activity ability. When the average crawling speeds of the IB and ds*GFP* beetles were 1.674 ± 0.122 cm/s and 1.818 ± 0.12 cm/s, respectively, the crawling speed of the ds*TnT* beetles was about 0.141 ± 0.033 cm/s (Figure 5E).

### 2.4. Identification of Differentially Expressed Genes

**Figure 6:**
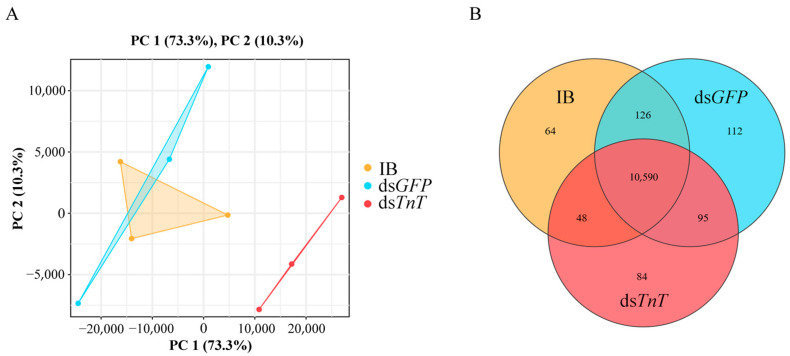
(**A**) Principal component analysis (PCA) of the samples. (**B**) A Venn diagram comparing the total numbers of genes.

RNA sequencing obtained 41,931,313, 43,866,045 and 39,546,558 clean reads in the IB, ds*GFP* and ds*TnT* groups, respectively. Uniquely mapped reads were all above 93%, while multiple mappings were all below 1.1% (Table S1), indicating that most of the reads from the nine samples were available for gene expression analysis. Principal component analysis showed that all three biological replicates of samples in the same treatment were highly correlated (Figure 6A). A total of 10,828, 10,923 and 10,817 genes were detected in the IB, ds*GFP* and ds*TnT* groups, respectively (Figure 6B).

**Figure 7:**
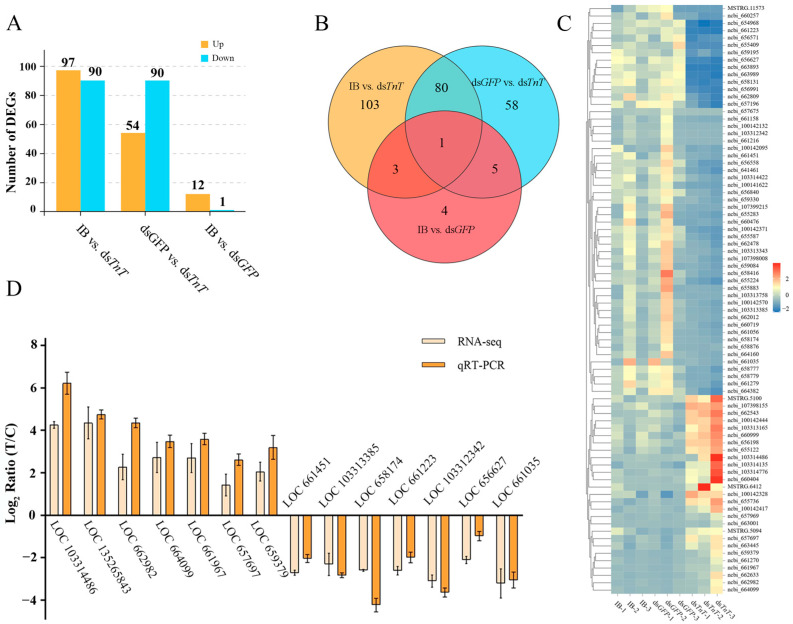
(**A**) The number of DEGs upregulated and downregulated in ds*TnT* relative to in the controls. (**B**) A Venn *diagram* comparing the DEGs. (**C**) Cluster heatmap analysis of 80 common DEGs from the comparison of the IB vs. ds*TnT* group and the comparison of the ds*GFP* vs. ds*TnT* group. Red means highly expressed genes; blue means low-expressed genes. (**D**) A comparative analysis of the expression level of the 14 randomly selected DEGs detected by RNA sequencing and qRT-PCR.

The differentially expressed genes (DEGs) were defined by a fold change > 2 and an FDR < 0.05, which screened out 187 DEGs in the IB vs. ds*TnT* group and 144 DEGs in the ds*GFP* vs. ds*TnT* group (Figure 7A). Compared to the IB group, 97 DEGs were upregulated and 90 DEGs were downregulated in the ds*TnT* sample. Compared to the ds*GFP* group, 54 DEGs were upregulated and 90 DEGs were downregulated in the ds*TnT* sample (Figure 7A). A Venn diagram and cluster heatmap showed that 80 DEGs were commonly shared in the IB vs. ds*TnT* group and ds*GFP* vs. ds*TnT* group, in which 27 DEGs were upregulated and 53 DEGs were downregulated (Figure 7B,C). These 80 genes are listed in Excel Table S2, found as additional material in a Supplementary File. Additionally, the expression patterns of the 14 randomly selected DEGs were similar between qRT-PCR and RNA sequencing (Figure 7D), indicating the reliability of the RNA-Seq results.

### 2.5. GO Classification and KEGG Analysis of All Detected Genes

**Figure 8:**
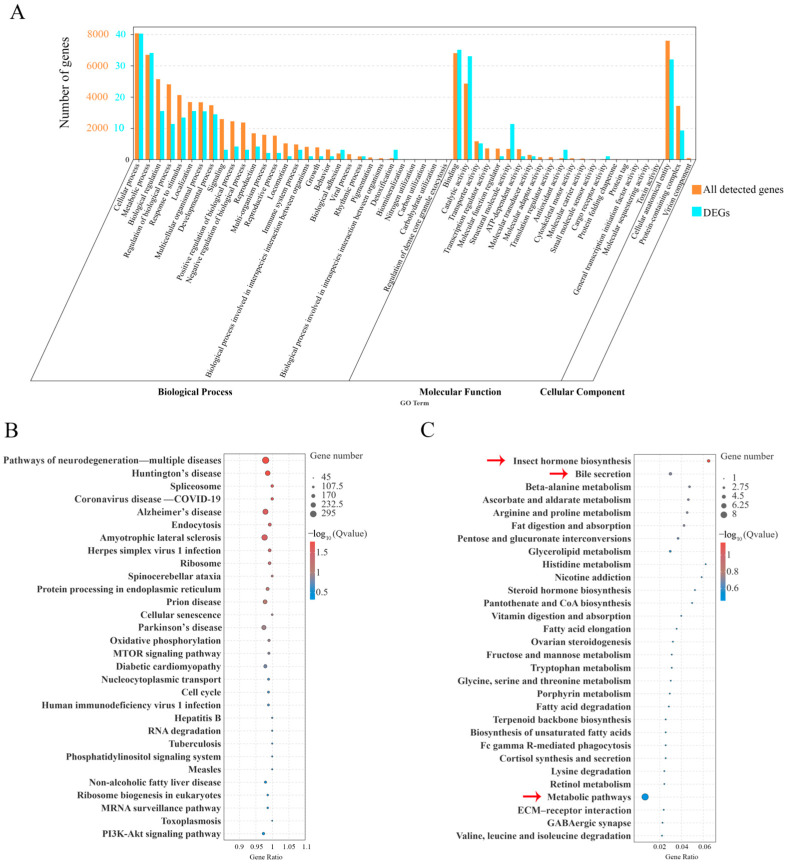
(**A**) Histogram of Gene Ontology (GO) classifications. The orange number on the *Y*-axis represents the number of all the detected genes, while the blue number represents 80 common DEGs from the comparison of the IB vs. ds*TnT* group and the comparison of the ds*GFP* vs. ds*TnT* group. (**B**) Scatter plot of the enriched Kyoto Encyclopedia of Genes and Genomes (KEGG) pathways for all the detected genes. (**C**) Scatter plot of the enriched KEGG pathways for 80 common DEGs. Size of the dots depends on the number of enriched genes; the color of the dots indicates pathways with significant enrichment (red) and low enrichment (blue). Gene ratio = the number of DEGs/the number of genes in this pathway. The top three enriched pathways were annotated with red arrows.

According to the GO functional analysis, all genes were enriched to 49 GO terms, and the 80 DEGs were enriched to 33 GO terms and classified into the categories of biological processes (BPs) with 22 GO terms, molecular function (MF) with 9 GO terms and cellular components (CCs) with 2 GO terms (*p* < 0.05) (Figure 8A). The top two enriched terms in BPs were cellular process (GO:0009987) and metabolic process (GO:0008152), including 72 DEGs in total (38.5% of this category), and the top two in MF were binding (GO: 0005488) and catalytic activity (GO:0003824), with 66 DEGs altogether (74.16% of this category), while the top enriched term in CCs was cellular anatomical entity (GO:0110165) with 31 DEGs (77.5% of this category) (Figure 8A). Among the 80 DEGs, several cuticle proteins and immune genes were significantly regulated. Data from the iBeetle-Base (https://ibeetle-base.uni-goettingen.de/, accessed on 20 October 2024) revealed that more than 20 DEGs would cause significantly lethal phenotypes after RNAi in *T. castaneum* (Table 1). More information about the GO enrichment of the genes is present in Excel Table S3, found as additional material in a Supplementary File.

Additionally, all genes were mapped to 352 reference pathways in the KEGG (Figure 8B), and the 80 DEGs were annotated in 59 biological pathways (Figure 8C). The top three enriched pathways (indicated by the red arrow in Figure 8C) were metabolic pathways (ko01100), insect hormone biosynthesis (ko00981) and bile secretion (ko04976), in which the expression levels of the DEGs belonging to the insect hormone biosynthesis pathway (ko00981) displayed the greatest difference. The specific genes were *farnesol dehydrogenase* (*FDH*), *aldehyde dehydrogenase family 3 member B1* (*ALDH3B1*) and *CYP314A1*. More information about the KEGG enrichment of the genes is shown in Excel Table S4, found as additional material in a Supplementary File.

## 3. Discussion

This study identified and comprehensively characterized the function of the *TnT* gene in the model insect *T. castaneum* with complete metamorphosis using RNAi and RNA sequencing. Based on the known basic functions of *TnT* in muscle function and locomotor mechanics, its indispensable roles in growth and development, nutrition metabolism, reproduction, etc., have been captured here. Since *TnT* is relatively highly conserved in insects and is distinctly different from that in vertebrates, RNAi targeting this gene would be highly efficient for insects and safe for non-target vertebrates, which means it may serve as a candidate target gene for the further creation and application of nucleic acid pesticides.

The continuous and relatively high expression of the *TnT* gene at all stages except for the embryonic stage implied that it may have a significant impact during the whole development period. The expression level reached a peak in the late pupae stage, in which a large number of the tissues and organs of the insect are degraded and reconstructed for metamorphosis. Therefore, after larval or early pupal RNAi treatment, *T. castaneum* could not complete its larval–pupal–adult transitions and had 100% cumulative mortality. During this process, the treatment caused abnormalities in the structure and function of multiple tissues (elytra, hindwings, ovaries, accessory gland, testes, gut), although the expression level of *TnT* in these tissues was not high. This was likely due to the deletion of *TnT* seriously disturbing the development of the muscle structure and proper muscle contraction in these tissues, which led to their dysfunction and in turn caused the beetles to die with a lower weight. In the incompletely metamorphosed insect *P. americana*, the RNAi of *TnT* caused severely atrophied wing phenotypes and completely blocked the molting process, which was highly consistent with our findings. It was speculated that wing muscles and whole-body muscle contraction were disturbed during molting after *TnT* knockdown [15]. However, *TnT* knockdown in *P. americana* had no effect on vitellogenesis or ovarian maturation but affected the peripheral muscles of the ovaries and then caused ovulation failure in over 75% of females, while the RNAi of *TnT* in *T. castaneum* seriously affected the morphology of the ovaries and embryonic formation, resulting in 100% inhibition of reproduction, which may have been due to the persistently high level of RNAi efficiency in the red flour beetle. Furthermore, the phenotype of a disrupted developmental process has also been found after the RNAi of other muscle structure-related genes. In *Rhynchophorus ferrugineus*, a higher mortality of 53.19% and lower weight were observed in larvae fed on actin dsRNA [18]. Injecting ds*TnI* resulted in a cumulative mortality of 85% in *Cylas formicarius* [19], while feeding on ds*TnI* led to the significant accumulation of food substances in the hindgut, finally causing a larval mortality of 94.6% [20]. Interestingly, the accumulation of flour also occurred at the mouthparts and midgut of *T. castaneum* in this study, which may have been related to the loss of peristaltic motion of the mandibular muscle and alimentary canal as well [3]. Defects in feeding and digestion can further cause nutritional deficiencies and metabolic abnormalities and decrease fecundity in insects. For example, defects in midgut protease genes in *Aedes aegypti* reduced the digestion of food, interrupted nutrient absorption and inhibited reproduction [21]. These phenotypes also appeared here in the ds*TnT*-treated beetles.

To further explore the molecular regulatory mechanism of *TnT*, a genome-wide transcriptomic analysis was performed on the fifth day after early pupal injection. Among the 80 common DEGs between the ds*TnT* group and the controls, several noticeably downregulated DEGs with biological significance for metamorphosis development were explored in relation to muscle function, nutrition metabolism, cuticle formation, and hormone signaling. *Actin* and *TnC*, which usually collaborated with *TnT* to maintain muscle function, were screened out. A total of three *glucose dehydrogenase genes* were significantly downregulated after *TnT* knockdown. Previous studies revealed that *glucose dehydrogenase* contributed to the degradation of the pupal cuticle in emergence preparation in *D. melanogaster*, and it also played important roles in carbohydrate metabolism, reproduction and immunity in insects [22,23,24,25]. Different types of enzyme genes related to nutrition metabolism, especially lipid metabolism (such as *lipase* and *fatty acyl-CoA reductase*), were also significantly downregulated, which could explain the phenotype of a dried and shriveled abdomen within a black-red, chunky fat body containing abnormal adipocytes in the ds*TnT*-treated adults. The downregulated cuticular proteins, such as larval cuticle protein A2B [26], adult-specific cuticular protein ACP-20 [27] and pupal cuticle protein 20 [28], were previously shown to affect cuticle formation and then intercept the ecdysis process, and they were regulated by 20E. Coincidentally, the mRNA levels of the key gene *CYP314* in the 20E synthesis pathway were significantly reduced by 81.47% in the ds*TnT* group, which may have directly caused the failure of metamorphosis development, accompanied by the emergence of the above phenotypes.

Troponin containing *TnT* was demonstrated as being a JH-responsive gene in insects. The knockdown of the JH receptor *Met* led to a significant decrease in the *TnT* mRNA level by 79.6% in the wing pads, and the injection of a JH analog into *P. americana* nymphs caused a 3.3-fold upregulated expression level of *TnT* mRNA [15]. In this study, the expression levels of *FDH* and *ALDH3B1*, two key enzymes oxidizing farnesol to farnesal and farnesoic acid, respectively, in the JH biosynthetic pathway [29,30], were significantly changed after ds*TnT* treatment, which may have been due to the effect of feedback regulation. JH signaling is necessary for regulating lipid storage, which determines free fatty acid homeostasis and triglyceride levels in the fat body during the insect growth and metamorphosis processes [31,32]. JH could also activate vitellogenin production in the fat body to undergo normal lipid metabolism and stimulate its uptake by maturing oocytes [33]. Additionally, it acts as a negative regulator of humoral immunity, mediating the immunity–reproduction trade-off [34]. Under the regulation of JH, muscle maintenance also plays indispensable roles in these processes [35,36]. Therefore, the appearance of abnormal adipocytes, a decreased reproductive ability and upregulated immune genes (*venom protease*, *protein slit-like*, *scavenger receptor class B member 1-like*) in the ds*TnT*-treated beetles may have been induced by muscle dysfunction and JH dysregulation.

## 4. Materials and Methods

### 4.1. Experimental Insects

Under standard conditions of 30 °C and 40% relative humidity, the GA-1 strain of *T. castaneum* was reared in 5% yeasted flour [37].

### 4.2. Phylogenetic Analysis

The *TnT* sequences of *T. castaneum* and other representative animals were obtained from NCBI (https://www.ncbi.nlm.nih.gov/, accessed on 10 September 2024). Phylogenetic analysis was performed based on the amino acid sequence alignment by the neighbor-joining method embedded in the MEGA 11 program. Finally, we visualized and annotated the results online using TVBOT (Version 2.6.1) (https://www.chiplot.online/tvbot.html, accessed on 10 September 2024). Motifs were analyzed using MEME (Version 5.5.7) (https://meme-suite.org/meme/tools/meme, accessed on 19 September 2024).

### 4.3. Quantitative Real-Time PCR Analysis

For the temporal and spatial expression profile analysis of TnT, total RNAs were extracted at the following developmental stages: early eggs (EEs, 1 day old), late eggs (LEs, 3 days old), early larvae (EL, 1 day old), late larvae (LL, last instar larvae), pre-pupae (PP, 1 day old); early pupae (EP, 1 day old), late pupae (LP, 5 days old), early adults (EAs, 1 day old) and late adults (LAs, 1 week old). We used a Total RNA Isolation Reagent (Vazyme, Nanjing, China). Approximately 50 mg samples were required for each egg stage and the early larval stages, while 3 individuals were used for each of the other stages. Additionally, total RNAs from various tissues of late adults (about 100 adults) were extracted: the tissues included the elytra, ovaries, accessory glands, gut, Malpighian tubule, hindwings, testes, central nervous system (CNS), fat body and integument. After the reverse transcription, quantitative real-time PCR (qRT-PCR) was performed to check the expression levels as described before. The data are expressed here as mRNA levels normalized to the stable house-keeping gene ribosomal protein S3 (rps3) in the corresponding cDNA sample, determined using the 2^−ΔΔCt^ method [38,39,40]. The primers are listed in Table S5.

### 4.4. RNA Interference (RNAi)

The cDNA fragments derived from the *TnT* sequence of *T. castaneum* and *green fluorescent protein* (*GFP*) were separately amplified by PCR using specific primers (Table S5). The dsRNA synthesis was performed with the TranscriptAid™ T7 High Yield Transcription Kit (Thermo Fisher Scientific, Waltham, MA, USA). Subsequently, the quality of dsRNAs was determined using the Nanodrop 2000 spectrophotometer (Thermo Fisher Scientific, Wilmington, NC, USA) and agarose gel electrophoresis, and they were diluted to a final concentration of 2 μg/μL and then kept at −80 °C [41]. Approximately 200 ng (100 nL) of ds*TnT* was injected into the fifth instar larvae or early pupae (1 day old). An equal volume of a buffer or ds*GFP* was used to inject larvae or early pupae in the negative control groups, named the IB group and ds*GFP* group, respectively. Every group had about 30 individuals. Three individuals were used to detect the RNAi efficiency on the third day after injection using qRT-PCR. Subsequently, the developmental status of the insects was observed under the same rearing conditions as mentioned above. Since *TnT* knockdown in the larval stage or early pupal stage caused 100% cumulative mortality before the adult stage, late pupal RNAi that would not cause any detectable defects in emergence was conducted to explore the effects of *TnT* on the body weight, reproduction and the locomotion ability in adults. The weight of adults (10 days old) in different groups was measured. For reproduction analysis, five groups were formed as follows: the IB, ds*GFP*, ds*TnT*, ds*TnT*♂ × IB♀ and ds*TnT*♀ × IB♂ groups. Single-pair mating (10 pairs in each group) was performed to check the fecundity of the beetles on the 10th day after eclosion. Furthermore, cameras were used to record the crawling of the adults (10 days old) on grid paper within a limited time, and then their motion trajectories were analyzed using Tracker software (Version 4.95, https://opensourcephysics.github.io/tracker-website/ accessed on 27 September 2024) to calculate the average crawling speed. At least three biological replications were performed for each experiment.

### 4.5. RNA Isolation and Sequencing

For RNA sequencing, dsRNA injection was carried out in early pupae (1 day old), because 100% of the pupae in the ds*TnT* group were blocked at a relatively consistent eclosion time. The total RNAs of individuals in different groups were extracted at the exact same eclosion time. After the quality and concentration detection, the mRNA was enriched, and the cleaved RNA fragments (approximately 200 bp) were used for first-strand cDNA synthesis using random hexamer primers. The double-stranded cDNA fragments were end-repaired, base-added, purified, and ligated to Illumina sequencing adapters. After being further purified and amplified, the Illumina Novaseq6000 by the Gene Denovo Biotechnology Co. (Guangzhou, China) was employed to sequence the resulting cDNA library.

### 4.6. Bioinformatics and Data Analysis

The raw data from the sequencing were submitted to the Short Read Archive (SRA) database of NCBI with the accession number PRJNA1171218. The clean reads were obtained from filtering the low-quality sequences in the raw reads and then aligned to the *T. castaneum* reference genome (https://www.ncbi.nlm.nih.gov/ accessed on 9 October 2024) using SOAPaligner/soap2 [42]. Further analysis was conducted on the obtained data for the functional annotation of all the detected genes. To quantify the expression abundance and variations, the fragments per kilobase of transcript per million mapped reads (FPKM) were calculated using RSEM software (Version 1.2.19). Differentially expressed genes (DEGs) and transcripts were identified based on an absolute fold change of > 2 and a false discovery rate (FDR) < 0.05. The obtained DEGs were searched for in iBeetle-Base (https://ibeetle-base.uni-goettingen.de/ accessed on 20 October 2024), with a primary focus on those genes that caused significant lethality phenotypes after RNAi. Finally, the mapping of all DEGs to Gene Ontology (GO) terms and pathway enrichment analysis were performed using the GO database (http://www.geneontology.org/ accessed on 15 November 2024) and the Kyoto Encyclopedia of Genes and Genomes (KEGG) database (https://www.genome.jp/kegg/ accessed on 28 November 2024), respectively.

### 4.7. Quantitative Real-Time PCR (qRT-PCR)

In order to verify the RNA sequencing results, the relative mRNA expression of 14 randomly selected DEGs was illustrated by qRT-PCR. Primers for these DEGs are shown in Table S6.

### 4.8. Statistical Analysis

The data were analyzed with a one-way analysis of variance (ANOVA) using SPSS version 27.0 software. All data were presented as the Mean ± SE, and *p* < 0.05 was regarded as statistically significant.

## 5. Conclusions

In summary, we identified *TnT*, a pivotal gene for growth and development, nutrition metabolism and reproduction in *T. cataneum*. We speculated that the RNAi of *TnT* induced the dysfunction of the muscle of several tissues, which then led to disorder in movement and the food intake, further resulting in the dysregulation of lipid metabolism by affecting the hormone levels, which ultimately completely inhibited the reproductive ability of and tremendously promoted mortality in the beetle. Our results suggest that *TnT* can be used as a potential target for pest control based on an RNAi strategy.

## Figures and Tables

**Table 1:** DEGs that would cause significant lethal phenotypes after RNAi (data from iBeetle-Base, accessed on 20 October 2024).

Gene ID	Log_2_ Ratio (TnT/IB)	Log_2_ Ratio (TnT/GFP)	Regulation	Protein	Phenotype After RNAi of DEGs in *Tribolium castaneum*
LOC659195	−1.18	−1.12	Down	troponin C	50.0% lethality ^(2)^
LOC641461	−1.2	−1.23	Down	L-ascorbate oxidase-like protein	100.0% lethality ^(1)^ 60.0% lethality ^(2)^
LOC658777	−1.39	−1.41	Down	progestin and adipoQ receptor family member 3	90.0% lethality ^(1)^
LOC664382	−1.48	−1.61	Down	pro-resilin	60.0% lethality ^(2)^
LOC660257	−1.51	−1.56	Down	elongation of very-long-chain fatty acid protein	100.0% lethality ^(1)^ 100.0% lethality ^(2)^
LOC655409	−1.74	−1.85	Down	actin, muscle	100.0% lethality ^(1)^ 100.0% lethality ^(2)^
LOC657196	−1.79	−1.85	Down	protein lethal (2) essential for life	50.0% lethality ^(1)^ 30.0% lethality ^(2)^
LOC658131	−1.98	−2.78	Down	DNA ligase 1	30.0% lethality ^(1)^; 30.0% lethality ^(2)^
LOC656558	−1.80	−1.91	Down	collagen alpha-1(V) chain	40.0% lethality ^(1)^; 40.0% lethality ^(2)^
LOC655283	−2.06	−1.90	Down	fatty acyl-CoA reductase wat	50% lethality ^(1)^
LOC658876	−2.25	−2.19	Down	splicing factor 3A subunit 2	90.0% lethality ^(1)^; 60.0% lethality ^(2)^
LOC655587	−2.30	−2.44	Down	peroxidase	50.0% lethality ^(1)^; 30.0% lethality ^(2)^;
LOC656840	−2.39	−2.68	Down	pupal cuticle protein 20	80% lethality ^(1)^
LOC661451	−2.43	−2.16	Down	cytochrome P450 CYP314A1	no/almost no eggs ^(1)^
LOC103313385	−2.48	−2.58	Down	general odorant-binding protein 70	60.0% lethality ^(1)^ 50.0% lethality ^(2)^
LOC661279	−2.63	−2.12	Down	aldehyde dehydrogenase family 3 member B1	40% lethality ^(2)^
LOC661223	−2.60	−2.67	Down	46 kDa FK506-binding nuclear protein	60% lethality ^(2)^
LOC103312342	−3.16	−3.23	Down	adult-specific cuticular protein ACP-20	20.0% lethality ^(2)^
LOC660476	−3.61	−3.15	Down	histidine-rich glycoprotein	50.0% lethality ^(1)^; head and thorax and abdomen segment random not present
LOC655883	−3.96	−4.25	Down	Osi2 DUF1676 domain-containing protein	40.0% lethality ^(1)^ 40.0% lethality ^(2)^
LOC103313758	−5.36	−5.11	Down	cyclin-dependent kinase inhibitor 1C	40.0% lethality ^(1)^; irregular larval musculature pattern ^(1)^
LOC107399215	−6.28	−5.82	Down	larval cuticle protein A2B-like	40.0% lethality ^(2)^

^(1)^ Lethality after 11 days of larval injection. ^(2)^ Lethality after 11 days of pupal injection.

## Data Availability

Raw sequence Data of the RNA-seq analysis in this study are available in the Short Read Archive (SRA) database of NCBI with the accession number PRJNA1171218 and NCBI URL as below: (PRJNA1171218, https://www.ncbi.nlm.nih.gov/sra/?term=PRJNA1171218).

## Supplementary Materials

The following supporting information can be downloaded at: https://www.mdpi.com/article/10.3390/ijms26062786/s1.
