# Supplementary material for: Identification and Characterization of Troponin T Associated with Development, Metabolism and Reproduction in Tribolium castaneum
Source: Int J Mol Sci. 2025 Mar 19;26(6):2786. doi: 10.3390/ijms26062786 (PMC11942869; doi:10.3390/ijms26062786)
Supplement: Supplementary file 1 [file ijms-26-02786-s001.zip › ijms-3470769-supplementary/Table S1 Summary of reads from the RNA sequencing data.pdf]

Table S1. Summary of reads from the RNA sequencing data

Sample name	Total reads	Total mapped reads	Unique matches	Multi-position matches	Total unmapped reads
IB_1	48775492	46751479 (95.85%)	46269174 (94.86%)	482305 (0.99%)	2024013 (4.15%)
IB_2	42997266	41108437 (95.61%)	40712644 (94.69%)	395793 (0.92%)	1888829 (4.39%)
IB_3	34021180	32304342 (94.95%)	31970731 (93.97%)	333611 (0.98%)	1716838 (5.05%)
ds*GFP*_1	36750380	34834525 (94.79%)	34479248 (93.82%)	355277 (0.97%)	1915855 (5.21%)
ds*GFP*_2	53436978	51006440 (95.45%)	50413695 (94.34%)	592745 (1.11%)	2430538 (4.55%)
ds*GFP*_3	41410778	39282457 (94.86%)	38875828 (93.88%)	406629 (0.98%)	2128321 (5.14%)
ds*TnT*_1	40875716	38874631 (95.10%)	38484795 (94.15%)	389836 (0.95%)	2001085 (4.90%)
ds*TnT*_2	42329226	40247702 (95.08%)	39855906 (94.16%)	391796 (0.93%)	2081524 (4.92%)
ds*TnT*_3	35434732	33568302 (94.73%)	33226522 (93.77%)	341780 (0.96%)	1866430 (5.27%)
