# Supplementary material for: Identification and Characterization of Troponin T Associated with Development, Metabolism and Reproduction in Tribolium castaneum
Source: Int J Mol Sci. 2025 Mar 19;26(6):2786. doi: 10.3390/ijms26062786 (PMC11942869; doi:10.3390/ijms26062786)
Supplement: Supplementary file 1 [file ijms-26-02786-s001.zip › ijms-3470769-supplementary/Table S5 Primers of TnT, GFP and the internal control gene rps3.pdf]

Table S5. Primers of *TnT*, *GFP* and the internal control gene *rps3*.

Gene name	Primer sequence (5'→3')	Application
TnT	F: GCTGACCTGGGGATAAGACAGT	qRT-PCR
	R: CTTGACGTTTGATGAATTCTGGAT	
rps3	F: TCAAATTGATCGGAGGTTTG	qRT-PCR
	R: GTCCCACGGCAACATAATCT	
TnT	F: TAATACGACTCACTATAGGGACCCAACTTCACCATC	RNAi
	R: TAATACGACTCACTATAGGGTTCTCGCTTGCTTATTC	
GFP	F: TAATACGACTCACTATAGGGTAAACGGCCACAAGTTCAG	RNAi
	R: TAATACGACTCACTATAGGGTCGGCCATGATATAGACGTT	
