# Supplementary material for: Identification and Characterization of Troponin T Associated with Development, Metabolism and Reproduction in Tribolium castaneum
Source: Int J Mol Sci. 2025 Mar 19;26(6):2786. doi: 10.3390/ijms26062786 (PMC11942869; doi:10.3390/ijms26062786)
Supplement: Supplementary file 1 [file ijms-26-02786-s001.zip › ijms-3470769-supplementary/Table S6 Primers used for qRT-PCR to validate the RNA-sequencing data.pdf]

Table S6. Primers used for qRT-PCR to validate the RNA-sequencing data

Accession number	Gene neame	Primer sequence (5'→3')
LOC662633	*probable cytochrome P450 4aa1*	F: CATTATCATGTTATGGACATCGTTT
		R: ACAAGCCGTGGCAAGTAGTAAA
LOC135265843	*synaptic vesicle glycoprotein 2B*	F: AGCCATATTCTCCTGTTGTTTTT
		R: GACGTCACCTGTTCCACCTC
LOC662982	*Protein slit-like Protein*	F: GCATCTCAGATATACCATAATTACGTG
		R: TCATTTTGACAGGAACCACGAC
LOC664099	*scavenger receptor class B, member 1*	F: TTTACTTGTTAGGCTTAGGTGGTTT
		R: GGGAATCGGGCTGTATTCG
LOC661967	*UDP-glucuronosyltransferase 2B2*	F: TCGCTTGATTTCCATTCGTTTA
		R: CTTATAACGTATTCAATCCACCACA
LOC657697	*chemosensory protein 20 precursor*	F: ATCCTCCAAAGTGACCGTCTCA
		R: GTTTTGGCTCCTTGTTTCTGCT
LOC659379	*dual oxidase maturation factor 1*	F: CAATTCGGACCAAATGGGC
		R: ACAAGCCGTGGCAAGTAGTAAA
LOC661451	*cytochrome P450 CYP314A1*	F: CCTAAACTACATCGCAGACCGA
		R: GGGAAGCCGAAGTAGGTGTCT
LOC103313385	*general odorant-binding protein*	F: CATTGCTGGTTGTTTGCTGC
		R: TCTTTCTGGGTCACTCCTTCG
LOC658174	*lipase member H-A*	F: GGGCACCAGAAGACTGAGAATAGA
		R: GCCGATAGGTAGCAGGGAAAC
LOC661223	*46 kDa FK506-binding nuclear protein*	F: GGAAGTGAAGCCGAAACAGAA
		R: CCTTTGCCAACCCTGAAACTAA
LOC103312342	*adult-specific cuticular protein ACP-20*	F: ATTTGGAAGAAGTTGGGTATGGG
		R: GCTTATCGGCGGTGTATTGG
LOC656627	*aspartyl/asparaginyl beta-hydroxylase*	F: GGGGCAAACCAAGTTCAGTG
		R: ATTAGGACAAGTCGGAGCGAAG
LOC661035	*sorbitol dehydrogenase*	F: TATTTGTGGCTCCGATGTCC
		R: ACAAGTGCGGCAAGTTATTCC
